# A Study of the Clinical Profiles of Patients With Hepatorenal Syndrome

**DOI:** 10.7759/cureus.66778

**Published:** 2024-08-13

**Authors:** Akshata Borle, Shubhangi Kanitkar, Prasad C Bagare, Muskaan Ahlawat, Sai Priya Ande

**Affiliations:** 1 Internal Medicine, Dr. D.Y. Patil Medical College, Hospital and Research Centre, Pune, IND; 2 Medicine, Dr. D.Y. Patil Medical College, Hospital and Research Centre, Pune, IND

**Keywords:** outcome, alcohol, hepatorenal, ascites, cirrhosis

## Abstract

Background and objective

Hepatorenal syndrome (HRS) is a systemic disorder that affects both the kidneys and the liver. HRS refers to the occurrence of kidney failure in individuals with advanced liver cirrhosis, portal hypertension, and ascites, without any underlying kidney disease. The interplay of systemic and portal hemodynamics causes severe constriction of blood vessels in the kidneys, which defines HRS. The study aims to illuminate the demographic profiles, etiology, and outcomes of patients with HRS.

Material and methods

The study was designed as a prospective, cross-sectional, hospital-based observational study conducted at Dr. D.Y. Patil Medical College, Hospital, and Research Centre in Pimpri, Pune. The study period spans from September 2022 to June 2024. Before commencement, approval was obtained from the institutional ethics committee, and informed written consent was secured from all participating patients. The sample size consists of 100 patients diagnosed with HRS, selected from the general medicine outpatient department, wards, and ICU of Dr. D.Y. Patil Hospital and Research Centre. A comprehensive clinical history was recorded for all patients, focusing on the symptoms of cirrhosis and HRS, followed by a thorough examination for related signs and symptoms.

Results

Among the 100 patients included in this study on HRS, 25% (N=25) fell within the age range of 18-30 years, and 76% (N=76) were identified as male. Alcoholic cirrhosis accounted for 78% (N=78) of cases, with hepatitis B infection being the subsequent leading cause. The mortality rate was 12% (N=12) while the survival rate was 88% (N=88).

Conclusion

This study provides insights into the demographic profile, etiology, and outcomes of HRS. The results of this study contribute valuable insights into the complex nature of HRS, highlighting the importance of early detection and monitoring to optimize patient care.

## Introduction

Hepatorenal syndrome (HRS) is a systemic disorder that impacts the liver and kidneys. The initial correlation between renal failure and cirrhosis was established in the late 1800s. In the mid to late 1900s, subsequent investigations revealed that renal failure in cases of liver cirrhosis was functional. This was observed in individuals diagnosed with HRS who did not exhibit proteinuria and had normal kidney histology [1].

Over time, the concept of HRS has evolved due to its diagnosis relying on several criteria and mostly being regarded as a diagnosis made after ruling out other possibilities. HRS is characterized by a deterioration in kidney function in persons with advanced cirrhosis. It is a very serious complication that occurs in people with advanced cirrhosis [2,3]. The 2015 International Club of Ascites study outlines specific diagnostic criteria for hepatorenal syndrome. HRS can be classified into two types based on the progression and severity of the disease. HRS-acute kidney injury (HRS-AKI), previously known as type 1, occurs suddenly as a result of liver failure and has a rapid and unfavorable prognosis. On the other hand, HRS-non-acute kidney injury (HRS-NAKI), referred to as type 2, progresses slowly and develops due to refractory ascites. Patients have reduced urine output, swelling, and unstable circulation due to significant dilatation of the blood vessels throughout the body [4].

The prevalence of HRS in individuals with decompensated liver disease is around 4%. The majority of these individuals have portal hypertension resulting from alcoholic hepatitis, cirrhosis, or metastatic malignancies. In individuals with decompensated liver disease, the likelihood of developing HRS within 1 year is 18%, and within 5 years is 39%. Patients with hyponatremia and high plasma renin activity had the greatest risk. Approximately 33% of individuals with spontaneous bacterial peritonitis have the potential to develop HRS [5].

In a prospective research by Bery A et al., the clinical profile of individuals with HRS was examined. A total of 42 patients were diagnosed with HRS and were included in the study. The prevalence of HRS was 0.275% among patients admitted to the hospital for medical reasons. Alcoholic cirrhosis was the cause in 71.5% of cases. The majority of HRS patients were given a mixture of dopamine, albumin, and terlipressin. The observed death rate was roughly 60%. In the non-survival group, oliguria and hepatic encephalopathy were more common. The non-survival group had greater levels of serum bilirubin, hypoalbuminemia, hyponatremia, coagulopathy, and urine osmolality [6].

Though there is various literature available on HRS, there is limited availability of data on the clinical profile of patients with HRS, especially in the given study area. Thus, this research was designed to study the clinical profile of patients with HRS.

Aims and objectives

Our study aims to illuminate the demographic profiles, etiology, and outcomes of patients with HRS.

## Materials and methods

Study design and setting

The present study was a prospective cross-sectional hospital-based observational study conducted at the Department of Medicine, Dr. D. Y. Patil Medical College, Hospital, and Research Centre, Pimpri, Pune. The study period extended from September 2022 to June 2024. Before the commencement of the investigation, approval was obtained from the Institute’s Scientific and Ethics Committee (ethical committee clearance number: IESC/PGS/2022/01). Written consent forms were provided to participants in their own languages, ensuring they understood the study’s goals, procedures, and potential risks.

Inclusion criteria

All patients above the age of 18 years who were diagnosed with HRS were included in the study. This criterion ensured that the study focused on adult patients with a confirmed diagnosis of HRS, providing a relevant population for analyzing the demographic profiles, causes, and outcomes associated with the syndrome.

Exclusion criteria

Patients with any form of structural renal pathology were excluded from the study. This exclusion criterion was implemented to ensure that the renal failure observed in the study participants was solely attributable to HRS and not influenced by any pre-existing kidney diseases or structural abnormalities.

Sample size

The sample size was calculated using WINPEPI software (http://www.brixtonhealth.com/pepi4windows.html), based on a prevalence rate of 59.6% for the development of HRS in cirrhotic patients with acute kidney injury, with a confidence level of 95% and an acceptable difference of 10%. The calculated sample size was 93; however, we conducted this study with a sample size of 100.

Data collection and consent

A detailed clinical history was taken from all patients using a pretested pro forma, focusing on the symptoms of HRS. We examined the patients for signs and symptoms of HRS and made both clinical and blood test diagnoses. Blood investigations included a complete blood count, serum electrolytes, renal function tests, liver function tests, international normalized ratio (INR), serum proteins, hepatitis B surface antigen (HBsAg), hepatitis C virus (HCV), HIV, urine routine microscopy, serum electrolytes, and urine sodium.

We rigorously followed the ethical approval and informed consent processes to ensure compliance with institutional and international standards. The collected data were meticulously analyzed to explore the clinical profile of patients with HRS.

## Results

Among the participants, 24 individuals were female, constituting 24% of the total sample. In contrast, 76 individuals were male, representing 76% of the total sample (Table 1).

**Table 1:** Distribution of gender among the study participants (N=100)

Gender	Frequency	Percentage
Female	24	24
Male	76	76

**Figure 1:**
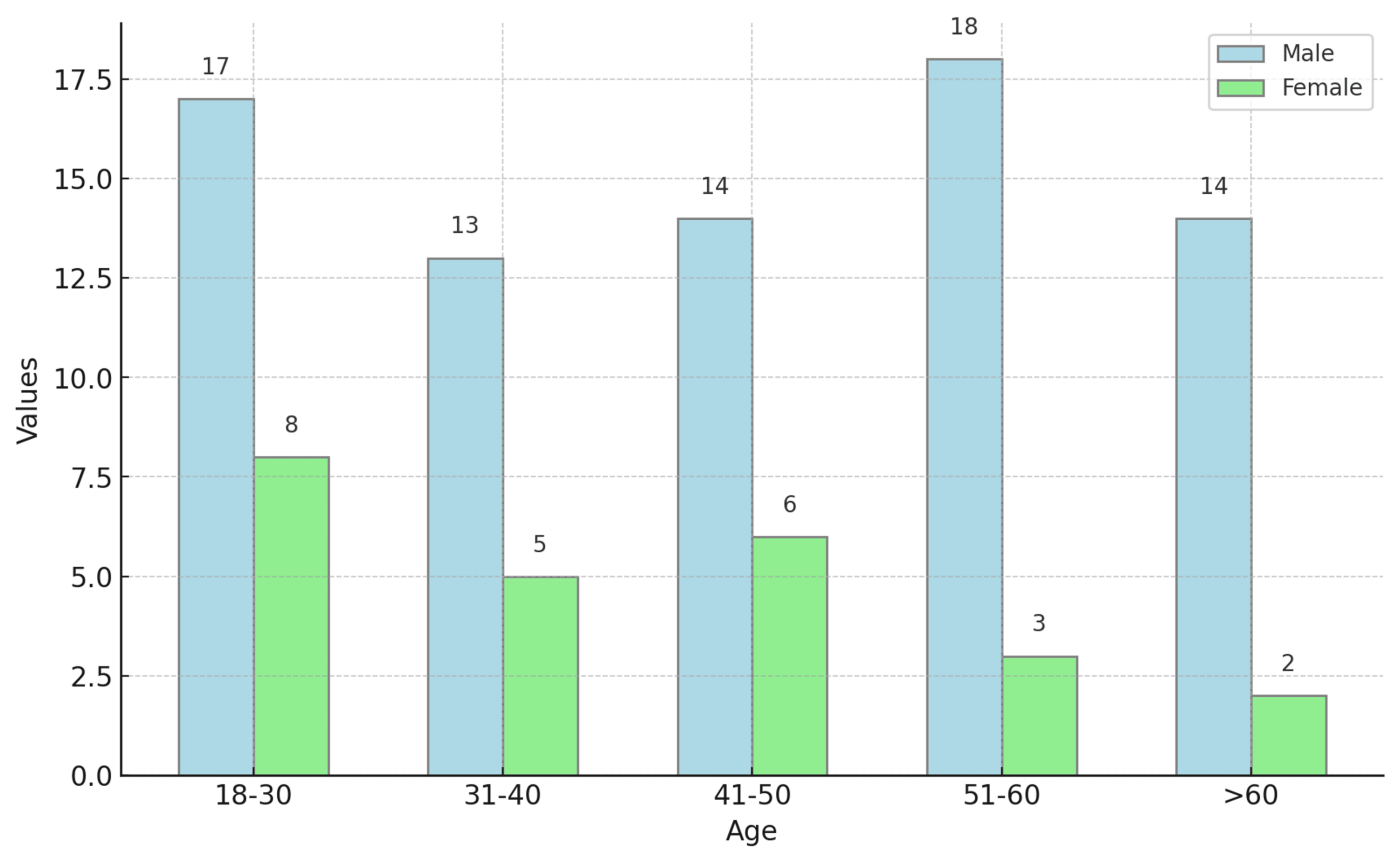
Distribution of age among the study participants (N=100)

The most common age group was 18-30 years (25%), followed by 51-60 years (21%), 41-50 years (20%), 31-40 years (18%), and >60 years (16%) (Table 2; Figure 1).

**Table 2:** Distribution of age among the study participants (N=100)

Age	Frequency	Male	Female	Percentage
18-30	25	17	8	25
31-40	18	13	5	18
41-50	20	14	6	20
51-60	21	18	3	21
>60	16	14	2	16

Alcoholic cirrhosis was the most prevalent etiology (78%), followed by hepatitis B virus (12%), non-alcoholic steatohepatitis (5%), hepatitis C virus (2%), and cryptogenic/other (3%) (Table 3).

**Table 3:** Distribution of etiology in male and female participants (N=100)

Cirrhosis etiology	Frequency	Male	Female	Percentage
Alcoholic	78	63	15	78
Hepatitis B Virus	12	7	5	12
Non-alcoholic steatohepatitis	5	2	3	5
Hepatitis C virus	2	1	1	2
Cryptogenic/other	3	3	0	3

Twelve percent (12%) of the study participants died during the hospital stay (Table 4).

**Table 4:** Distribution of outcomes during hospital stay among the study participants (N=100)

Outcome	Frequency	Percentage
Death	12	12
Alive	88	88

Our study analyzed the association of serum creatinine, serum sodium, and urine sodium with the outcome.

The serum creatinine levels were similar between the two groups, with a mean of 2.28 ± 1.08 mg/dL in the deceased group and 2.34 ± 0.93 mg/dL in the survivors, showing no significant difference (p = 0.84). This suggests that serum creatinine levels did not have a significant impact on the survival outcomes in this dataset.

The serum sodium levels for patients with a value less than 135 mEq/L had a mean of 129.92±2.39 mEq/L in the deceased group. A P value of 0.42 suggests that low serum sodium levels did not have a significant impact on survival outcomes.

There is a significant difference in urinary sodium levels between the two groups. The mean urinary sodium was significantly lower in the deceased group (3.34 ± 0.45 mmol/L) compared to the survivors (8.34 ± 0.75 mmol/L), with a p-value of less than 0.001. This highly significant difference suggests that lower urinary sodium levels are strongly associated with higher mortality in the studied population (Table 5).

**Table 5:** Association of laboratory parameters with outcomes among the study participants (N=100)

Variable	Death	Alive	p
Serum creatinine mg/dL	2.28±1.08	2.34±0.93	0.84
Serum sodium mEq/L <135	129.92±2.39	128.83±4.62	0.42
Urinary sodium mmol/L	3.34±0.45	8.34±0.75	<0.001

## Discussion

This study examines the demographic characteristics, etiology, and consequences of people with HRS. The study involved a group of 100 patients who were diagnosed with HRS. Our research revealed that 25% of the patients were within the age range of 18-30 years; however, the majority of them (59%) were between the ages of 31 and 60 years. Our study found that 76% of the people diagnosed with HRS were male, whereas 24% were female. The gender distribution in our study is consistent with the results reported by Bery et al., who also found a similar male predominance [6]. The fundamental cause for the higher prevalence of liver disease and alcohol consumption among males is often attributed to the greater frequency of alcohol intake, which in turn leads to a higher number of male patients with HRS. Janicko et al. did a subsequent investigation, utilizing a study sample with a mean age of 46.3±11.5. Most of the participants in the study were male [7]. This corresponds to the age distribution reported in previous research, which also demonstrated that HRS mainly affects adults in their middle age. For example, a study conducted by Roula Sasso et al. discovered that HRS tends to impact individuals who are of a similar age. This suggests that HRS is frequently seen in persons who have been exposed to risk factors such as prolonged alcohol usage and chronic liver disease [8]. Janicko et al. conducted a study that found the average age of participants to be 46.3±11.5, with a majority of them being male [7]. Salerno et al. conducted a study with a group of 134 patients who were diagnosed with cirrhosis. The average age of the patients was 61.3 years, with a standard variation of 9.6 years. Moreover, the study revealed that 73% of the participants were classified as male [9]. Alcoholic cirrhosis was the predominant etiology of HRS in our study, including 78% of patients, with hepatitis B accounting for 12%. This aligns with the results of Bery et al., in which alcoholic cirrhosis accounted for 71.5% of instances of HRS. The significant occurrence of alcoholic cirrhosis in both groups emphasizes the urgent requirement for efficient public health initiatives to decrease alcohol intake and avert liver disease [6]. However, the study conducted by Heidemann et al. found that alcohol consumption and cryptogenic cirrhosis were the main factors responsible for cirrhosis in patients with HRS [10]. Regarding laboratory results, our study discovered no notable disparity in serum creatinine levels between individuals who survived and those who did not, which contradicts the findings of Bery et al., who observed that increased serum creatinine was linked to unfavorable outcomes [6]. Nevertheless, our investigation revealed a noteworthy disparity in urine sodium concentrations, where lower levels exhibited a robust correlation with increased mortality (p < 0.001). This indicates that urine sodium may be a more accurate indicator for predicting the prognosis of HRS, which is consistent with the results of various other research that have identified low urinary sodium levels as a predictor of unfavorable outcomes in HRS patients. Hinz M. et al. conducted a study with similar parameters and discovered a link of similar magnitude between decreased sodium levels in urine and unfavorable survival outcomes [11]. The mortality rate in our study was 12%, whereas the survival rate was 88%. This is markedly lower than the 60% fatality rate documented by Bery et al. [6]. The variation observed may stem from disparities in research methodology, demographics of the patients involved, or the healthcare environments in which the studies were conducted. The reduced mortality rate seen in our study suggests that our healthcare establishment has implemented more effective management strategies and early intervention for HRS.

Limitations

The study provides valuable insights into the demographic profile, etiology, and outcomes of patients with HRS. However, several limitations should be acknowledged. One significant limitation is that the study was conducted at a single medical institution, Dr. D.Y. Patil Medical College Hospital and Research Centre in Pimpri, Pune. This limits the findings' generalizability to other regions or healthcare settings with different patient demographics and healthcare practices.

Another notable limitation is the absence of a control group of patients without HRS. This lack of a control group limits our ability to compare outcomes and identify specific factors that are uniquely associated with HRS. Without this comparison, it is difficult to draw definitive conclusions about HRS's distinct characteristics and prognostic factors compared to other conditions.

## Conclusions

Our study provides valuable insights into the demographic profile, etiology, and outcomes of patients with HRS. The predominance of alcoholic cirrhosis as a cause of HRS underscores the need for targeted interventions to reduce alcohol-related liver disease. Additionally, the significant association between low urinary sodium levels and mortality emphasizes the need for close monitoring of this parameter in HRS patients. These findings contribute to the broader understanding of HRS and can help inform future research and clinical practice.

The study highlights the importance of early detection and monitoring to improve patient care for those with HRS. Despite the limitations, the results provide a foundation for future studies and potential improvements in clinical strategies. The insights gained emphasize the critical role of addressing modifiable risk factors, such as alcohol consumption, and highlight urinary sodium levels as a crucial prognostic factor in managing HRS.
